# Supplementary material for: Transcriptional Response to Hypoxia: The Role of HIF-1-Associated Co-Regulators
Source: Cells. 2023 Mar 3;12(5):798. doi: 10.3390/cells12050798 (PMC10001186; doi:10.3390/cells12050798)
Supplement: Supplementary file 1 [file cells-12-00798-s001.zip › Figure S1.pdf]

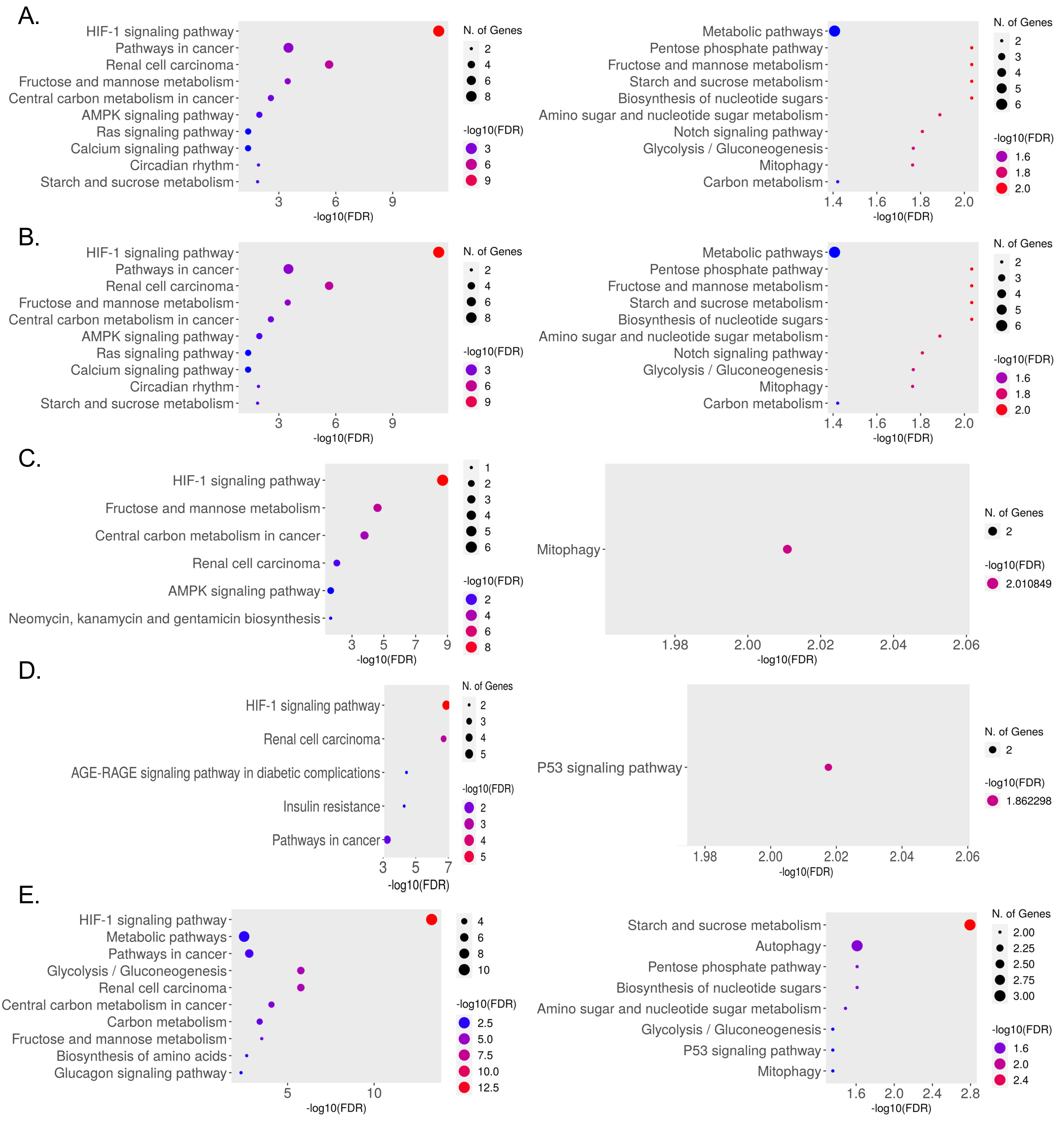

Supplementary Figure S1. Dot plots of KEGG pathway analysis of genes common between gene sets regulated by ZMYND8 (A), CDK8 (B), JMJD1A (C), NPM1 (D) or TRIM28 (E) and List A (left panels) or List B (right panels) genes.
